# Population impacts of conditional financial incentives and a male‐targeted digital decision support application on the HIV treatment cascade in rural KwaZulu Natal: findings from the HITS cluster randomized clinical trial

**DOI:** 10.1002/jia2.26248

**Published:** 2024-05-02

**Authors:** Maxime Inghels, Hae‐Young Kim, Thulile Mathenjwa, Maryam Shahmanesh, Janet Seeley, Sally Wyke, Philippa Matthews, Oluwafemi Adeagbo, Dickman Gareta, Nuala McGrath, H. Manisha Yapa, Ann Blandford, Thembelihle Zuma, Adrian Dobra, Till Bärnighausen, Frank Tanser

**Affiliations:** ^1^ Lincoln International Institute for Rural Health University of Lincoln Lincoln UK; ^2^ Centre Population et Développement (UMR 196 Paris Descartes – IRD), SageSud (ERL INSERM 1244) Institut de Recherche pour le Développement Paris France; ^3^ Africa Health Research Institute KwaZulu‐Natal South Africa; ^4^ Department of Population Health New York University School of Medicine New York City New York USA; ^5^ Institute for Global Health University College London London UK; ^6^ Department of Global Health and Development London School of Hygiene and Tropical Medicine London UK; ^7^ School of Social and Political Sciences, School of Health and Wellbeing University of Glasgow Glasgow UK; ^8^ Department of Sociology University of Johannesburg Johannesburg South Africa; ^9^ Department of Community and Behavioral Health College of Public Health University of Iowa Iowa City Iowa USA; ^10^ School of Primary Care, Population Sciences and Medical Education, Faculty of Medicine University of Southampton Southampton UK; ^11^ Department of Social Statistics and Demography, Faculty of Social Sciences University of Southampton Southampton UK; ^12^ Westmead Clinical School, Faculty of Medicine & Health University of Sydney Sydney New South Wales Australia; ^13^ University College London Interaction Centre University College London London UK; ^14^ University of Washington Seattle Washington USA; ^15^ Heidelberg Institute of Global Health (HIGH) Heidelberg University Heidelberg Germany; ^16^ Centre for the AIDS Programme of Research in South Africa (CAPRISA) University of KwaZulu‐Natal Durban South Africa; ^17^ Centre for Epidemic Response and Innovation, School for Data Science and Computational Thinking Stellenbosch University Stellenbosch South Africa; ^18^ The South African Centre for Epidemiological Modelling and Analysis (SACEMA) Stellenbosch University Stellenbosch South Africa

**Keywords:** HIV, HIV care cascade, home‐based HIV testing, financial incentives, counselling, mHealth

## Abstract

**Introduction:**

In South Africa, the HIV care cascade remains suboptimal. We investigated the impact of small conditional financial incentives (CFIs) and male‐targeted HIV‐specific decision‐support application (EPIC‐HIV) on the HIV care cascade.

**Methods:**

In 2018, in uMkhanyakude district, 45 communities were randomly assigned to one of four arms: (i) CFI for home‐based HIV testing and linkage to care within 6 weeks (R50 [US$3] food voucher each); (ii) EPIC‐HIV which are based on self‐determination theory; (iii) both CFI and EPIC‐HIV; and (iv) standard of care. EPIC‐HIV consisted of two components: EPIC‐HIV 1, provided to men through a tablet before home‐based HIV testing, and EPIC‐HIV 2, offered 1 month later to men who tested positive but had not yet linked to care. Linking HITS trial data to national antiretroviral treatment (ART) programme data and HIV surveillance programme data, we estimated HIV status awareness after the HITS trial implementation, ART status 3 month after the trial and viral load suppression 1 year later. Analysis included all known individuals living with HIV in the study area including those who did not participated in the HITS trial.

**Results:**

Among the 33,778 residents in the study area, 2763 men and 7266 women were identified as living with HIV by the end of the intervention period and included in the analysis. After the intervention, awareness of HIV‐positive status was higher in the CFI arms compared to non‐CFI arms (men: 793/908 [87.3%] vs. 1574/1855 [84.9%], RR = 1.03 [95% CI: 0.99−1.07]; women: 2259/2421 [93.3%] vs. 4439/4845 [91.6%], RR = 1.02 [95% CI: 1.00−1.04]). Three months after the intervention, no differences were found for linkage to ART between arms. One year after the intervention, only 1829 viral test results were retrieved. Viral suppression was higher but not significant in the EPIC‐HIV intervention arms among men (65/99 [65.7%] vs. 182/308 [59.1%], RR = 1.11 [95% CI: 0.88−1.40]).

**Conclusions:**

Small CFIs can contribute to achieve the first step of the HIV care cascade. However, neither CFIs nor EPIC‐HIV was sufficient to increase the number of people on ART. Additional evidence is needed to confirm the impact of EPIC‐HIV on viral suppression.

## 1 INTRODUCTION

Despite progress towards UNAIDS's 95‐95‐95, the number of people living with HIV (PLHIV) aware of their HIV status or on antiretroviral treatment (ART) remains insufficient in South Africa [1]. While the 2016 universal test and treat policy expanded access to ART, male initiation lags behind at 68%, compared to 80% for women [1]. Proactive interventions like home‐based HIV testing are effective in increasing awareness and linkage to ART, but they alone are insufficient to reach the UNAIDS target [2, 3]. To improve the impact of those interventions, several studies have investigated the effect of conditional financial incentives (CFIs) [4, 5, 6]. CFI can significantly increase testing uptake and new HIV diagnosis [4, 5, 6], but its effect on ART linkage and viral suppression remains mixed [7, 8, 9, 10]. Unlike testing uptake, ART linkage precedes ART retention, which reflects long‐term engagement with healthcare. ART linkage and retention mainly rely on intrinsic motivation that can be stimulated by health knowledge and empowerment [11]. Interventions like counselling, which support health knowledge and decision‐making, have been shown to be effective in improving linkage to care after home‐based HIV testing [2, 12, 13]. Capitalizing on the growth of mobile phone use in Africa, many of these interventions are now being adapted into mHealth versions [14]. Yet, many of these interventions have not been properly assessed in controlled clinical trials in sub‐Saharan Africa [2]. In addition, the impact of CFI or decision support application on the HIV care cascade using an intention‐to‐treat approach at a population level (i.e. including non‐participants residing in the area) remains undocumented.

Results of the “Home‐Based Intervention to Test and Start” (HITS) clinical trial showed a significant increase of home‐based HIV testing uptake in arms with CFI, an increase of early linkage to care (i.e. within 6 weeks) among women in the CFI arms but no effect of CFI or male‐targeted HIV‐specific decision‐support application (EPIC‐HIV) on early linkage to care of men [15, 16, 17, 18]. In this paper, we leverage more than 20 years of an HIV surveillance programme to describe the overall contribution of the two interventions (CFI and EPIC‐HIV) on HIV status awareness and ART linkage among all PLHIV residing in the HIV surveillance programme area. Additionally, we investigate the effects at 1 year of these interventions on achieving community viral suppression.

## 2 METHODS

### 2.1 Design

Between February and December 2018, we conducted a 2x2 factorial design cluster‐randomized clinical trial in 45 communities (i.e. group of households) in the Hlabisa sub‐district of the uMkhanyakude district of northern KwaZulu‐Natal, South Africa [19]. The trial was designed to measure the impact of two interventions, CFI and a male‐targeted HIV‐specific decision support called EPIC‐HIV (Empowering People through Informed Choices for HIV), on home‐based HIV testing uptake and linkage to care [20, 21]. Each community (i.e. cluster) was randomly assigned to one of the following arms: (i) CFI, 8 clusters, (ii) EPIC‐HIV, 8 clusters, (iii) CFI and EPIC‐HIV, 8 clusters and (iv) Standard of care, 21 clusters. The full trial protocol is published elsewhere [22].

The trial is nested within the demographic and HIV surveillance system led by the Africa Health Research Institute (AHRI) [23]. Since 2003, an annual population‐based HIV surveillance round collects blood samples from all residents aged 15 years and older in the study area after obtaining written informed consent. Since 2017, rapid HIV testing with immediate result has been offered during household visits. The population‐based HIV surveillance is linked with the clinical records of patients enrolled in HIV Treatment and Care Programme at the district hospital and 17 primary healthcare clinics using the Tier.Net electronic record system [24] and, since 2017, to the AHRILink which records all patients’ visits to the 11 clinics located in the study area.

### 2.2 Inclusion criteria

Every resident of 15 years of age and over who agreed to participate in the annual HIV surveillance was eligible to participate in the trial. Those who reported already being on ART were not eligible for any intervention.

### 2.3 Control arm

In the control arm, participants were offered home‐based rapid HIV testing with immediate result delivery. Individuals who tested positive were referred to the nearest HIV clinic.

### 2.4 Intervention arms

In the CFI arm, participants were offered a micro‐incentive in the form of a R50 food voucher (∼3 US$) conditional on accepting the home‐based rapid HIV test. If tested positive, participants were offered a second micro‐incentive of the same amount conditional on being linked to care within 6 weeks following the HIV test.

In the EPIC‐HIV arm, male participants were offered a tablet‐based application EPIC‐HIV 1 to support their decision to undergo the home‐based rapid HIV test. If they tested positive, men who were not linked to care within 1 month following the HIV test were re‐visited by the study‐tracking team who offered EPIC‐HIV 2 [20], which was designed to address any barriers for linkage to care and encourage linkage to care.

In the combined arm, male participants received EPIC‐HIV 1 prior to home‐based rapid HIV testing and both male and female participants received the R50 food voucher if they agreed to do the test. In cases of a positive result, participants received a R50 food voucher conditional on being linked to care within 6 weeks following the HIV test. Men with a positive result were also offered EPIC‐HIV 2 if they were not linked into care within 1 month following the HIV test.

### 2.5 Outcomes

People were defined as living with HIV if they met one of the following conditions: (i) had a positive HIV test during any previous population‐based HIV surveillance round, (ii) ever documented as being on ART in the Tier.Net database, (iii) ever had an ART visit documented in the AHRILink database or (iv) self‐reported as living with HIV during the HIV surveillance.

PLHIV were considered aware of their status if they met one of the following conditions: (i) had a documented ART appointment in the Tier.Net or AHRILink database, (ii) self‐reported as living with HIV, (iii) had a documented positive rapid test with immediate results and (iv) self‐reported having done an HIV test occurring after the earlier date of documented HIV‐positive status. The earlier date of documented HIV‐positive status was defined by taking the earlier date for the following events: earlier date of positive test results or self‐report of living with HIV documented in the population‐based HIV surveillance, earlier date of ART initiation documented in Tier.Net or AHRILink database. Self‐reported last HIV testing date was collected during previous HIV surveillance round (i.e. “When was your last test results? Less than six months; six months to one year ago; more than a year ago”), we then estimated the date of the last test by taking the right endpoint for the two first intervals (i.e. we considered 6 months if participant answer less than 6 months and 1 year for those would answered between 6 months and 1 year ago). Among the 374 PLHIV documented with only “more than a year ago” answers for their last tests, 372 ever self‐reported living with HIV or had a documented ART visit prior to the trial and were considered as aware of their results. The remaining two individuals were assumed to be not aware of their result.

Individuals living with HIV were considered on ART if they were documented as on ART in the Tier.Net datasets or have a recent ART appointment (less than 3 months) in the AHRILink datasets.

As part of the annual population‐based HIV surveillance, viral load (VL) testing is conducted for all collected dried blood samples that are tested positive for HIV. The detectable limit of the assay used on dried blood spots was 1550 copies/ml.

### 2.6 Statistical analysis

Using HIV surveillance datasets, HIV status was ascertained for all resident after the trial completion. All individuals identified as living with HIV were included in our analysis, irrespective of their participation in the trial. The impact of the trial interventions was measured by comparing pre‐trial baseline (i.e. up until the HITS interventions) outcomes with those assessed after completion of the trial interventions (HIV status awareness), 3 months later (ART status) and 1 year later (viral suppression).

Effects of CFI and EPIC‐HIV on each step of the HIV care cascade were analysed by intervention groups and factorial analysis. We used a modified Poisson regression model with a logarithm link function adjusted for community‐level clustering and binary outcomes through clustered sandwich estimators [25, 26]. To address the imbalance in sex and age distribution among PLHIV participants who provided blood samples for VL testing during the 2018 or 2019 surveys, we applied a post‐stratification weight based on age and sex to the VL‐related results (Appendix S1).

For the HIV status awareness outcome, we conducted sensitivity analysis by taking the mid‐point for the self‐reported last HIV testing date interval.

Analyses were stratified by sex. All analyses were conducted in R 4.2.2. with the packages *sandwich* for the models estimators and related confidence intervals and the package *survey* for the cluster‐adjusted confidence interval of descriptive results [27, 28].

### 2.7 Role of the funding source

The funders of the study had no role in study design, data collection, data analysis, data interpretation or writing of the article.

## 3 RESULTS

### 3.1 Flow chart description of the trial

Overall, we enumerated a population of 37,028 individuals in 2017, the year before the HITS intervention was implemented. Among them, 3250 were excluded because they were deceased or migrated out of the study area, leading to 33,778 resident individuals who were eligible to participate.

Among them, 24.2% (8188/33,778) were not contacted (due to not being found at home after three attempts or temporarily away for more than 2 months) and 29.3% (9910/33,778) refused to participate in the annual HIV surveillance survey (Appendix S2). Overall, 46.4% (15,680/33,778) of the residents participated in the trial—enrolment was 35.1% (4875/13,893) among men and 54.3% (10,805/19,885) among women.

### 3.2 HIV status ascertainment and population included

**Figure 1:**
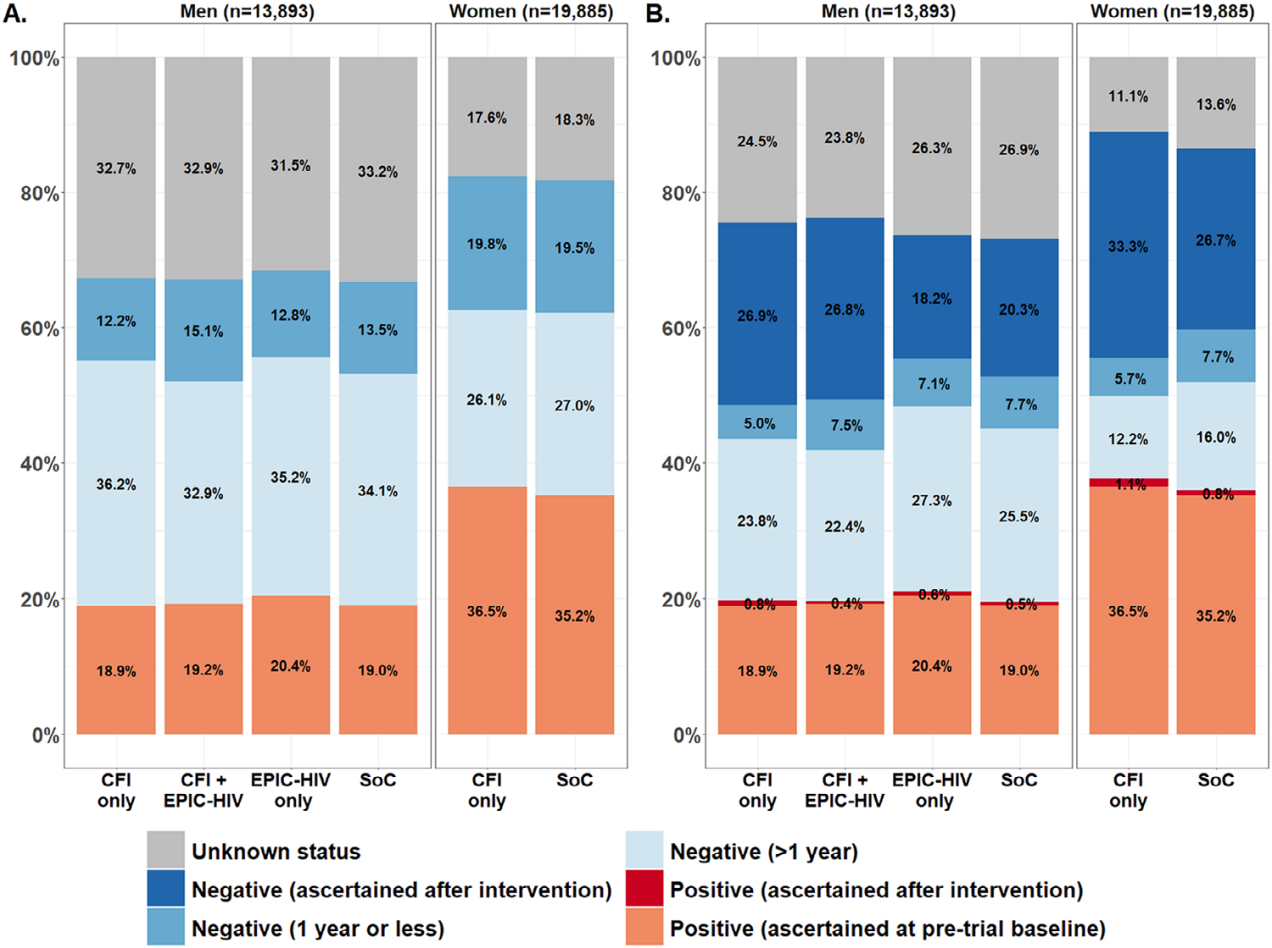
Documented HIV status at pre‐trial baseline (A) and after intervention (B) per trial arm, 2018 (*n* = 33,778). CFI, conditional financial incentive; EPIC‐HIV, Empowering People through Informed Choices for HIV; HITS, home‐based intervention to test and start; SoC, standard of care. Note 1: Unknown status includes indeterminate results. Note 2: Positive status ascertained after intervention includes both those tested by home‐based rapid test and those who agree to provide a blood sample for the HIV surveillance survey.

Using the HIV surveillance data, HIV status after trial completion was ascertained for 81.8% (27,634/33,778) of the trial‐eligible individuals residing in the study area (74.1% [10,300/13,893] among men and 87.2% [17,334/19,885] among women) (Figure 1). At pre‐trial baseline, the percentage of recently ascertained HIV‐negative individuals was non‐significantly different in the arms with CFI compared to those without CFI (respectively, 13.5% [625/4624] vs. 13.4% [1238/9269] among men, *p* = 0.90 and 19.8% [1276/6433] vs. 19.5% [2623/13,452] among women, *p* = 0.87). The percentage of recently ascertained HIV‐negative persons after the trial completion was higher in the arms with CFI compared to those without CFI (respectively, 26.9% [1243/4624] vs. 19.7% [1830/9269] among men, *p*<0.001 and 33.3% [2142/6433] vs. 26.7% [3588/13452] among women, *p*<0.01).

Overall, 2763 men and 7266 women were ascertained as living with HIV were included in our analysis. Among them, 434 (96 men and 338 women) and 58 (13 men and 45 women), respectively, benefited from a CFI by accepting home‐based HIV‐testing and being linked to care within 6 weeks. Only 122 and 14 men completed the EPIC‐HIV‐1 and EPIC‐HIV‐2 application, respectively.

### 3.3 HIV‐positive status awareness

**Figure 2:**
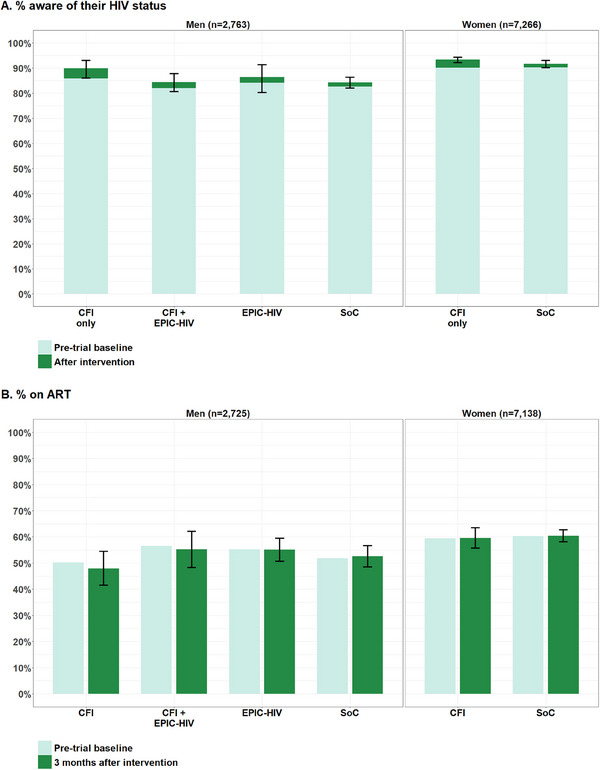
Percentage of men and women living with HIV aware of their status (A) and on ART (B) per trial arm, 2018. ART, antiretroviral treatment; CFI, conditional financial incentive; EPIC‐HIV, Empowering People through Informed Choices for HIV; SoC, standard of care. Note 1: In Figure A, 95% confidence intervals are computed for the HIV status awareness after intervention. Note 2: Total headcounts for linkage to ART before the HITS visit date are 2725 for men and 7138 for women. Note 3: For some arms, the percentage on ART was found lower after 3 months due to individuals interrupting care, dying or moving out of the study area.

Overall, 85.7% [95% CI 84.0–87.0] (2367/2763) of men and 92.2% [91.1–93.0] (6698/7266) of women living with HIV were ascertained as aware of their status after the intervention (Figure 2A). No significant differences were observed between arms at pre‐trial baseline (global *p*‐value, *p* = 0.45 and *p* = 0.67 among men and women, respectively). When considering only the 122 men and 375 women who tested positive by rapid HIV test during the trial visit, respectively, 53.3% and 40.3% of them were not previously aware of their HIV‐positive status.

Overall, residents in the CFI arms had a significantly higher probability of being aware of their HIV‐positive status (RR 1.02 [1.00–1.04], *p* = 0.03). In subgroup analyses, this effect remained positive among women and men, although this association did not reach statistical significance for men (Table 1). The EPIC‐HIV application had no effect on HIV status awareness among men (RR 1.00 [0.96–1.04], *p* = 0.90). The analysis by taking the date of last HIV test by midpoint method showed similar risk ratios (Appendices S3 and S4).

**Table 1:** Risk factors for HIV status awareness, linkage to ART and viral load suppression following HITS intervention per arm, 2018−2019 (*n* = 10,029)

	Men (*n* = 2763)			Women (*n* = 7266)		
	*n*/*N* (%)	Relative risk (95% CI)	*p*‐value	*n*/*N* (%)	Relative risk (95% CI)	*p*‐value
HIV status awareness						
Per arm						
CFI only	442/492 (89.8)	1.07 [1.03−1.11]	0.001	2259/2421 (93.3)	1.02 [1.00−1.04]	0.042
CFI + EPIC‐HIV	351/416 (84.4)	1.00 [0.96−1.04]	0.934	n/a	n/a	
EPIC‐HIV only	458/530 (86.4)	1.03 [0.97−1.08]	0.365	n/a	n/a	
Standard of care	1116/1325 (84.2)	*ref*		4439/4845 (91.6)	*ref*	
Per CFI arms						
CFI	793/908 (87.3)	1.03 [0.99−1.07]	0.134	2259/2421 (93.3)	1.02 [1.00−1.04]	0.042
No CFI	1574/1855 (84.9)	*ref*		4439/4845 (91.6)	*ref*	
Per EPIC‐HIV arms						
EPIC‐HIV	809/946 (85.5)	1.00 [0.96−1.04]	0.896	n/a	n/a	
No EPIC‐HIV	1558/1817 (85.7)	*ref*		n/a	n/a	
ART status (3 months after intervention)^a^						
Per arm						
CFI only	233/486 (47.9)	0.91 [0.80−1.04]	0.162	1425/2389 (59.6)	0.99 [0.92−1.06]	0.725
CFI + EPIC‐HIV	226/409 (55.3)	1.05 [0.93−1.19]	0.437	n/a	n/a	
EPIC‐HIV only	289/524 (55.2)	1.05 [0.95−1.15]	0.338	n/a	n/a	
Standard of care	687/1306 (52.6)	*ref*		2869/4749 (60.4)	*ref*	
Per CFI arms						
CFI	459/895 (51.3)	0.96 [0.87−1.06]	0.437	1425/2389 (59.6)	0.99 [0.92−1.06]	0.725
No CFI	976/1830 (53.3)	*ref*		2869/4749 (60.4)	*ref*	
Per EPIC‐HIV arms						
EPIC‐HIV	515/933 (55.2)	1.08 [0.99−1.17]	0.094	n/a	n/a	
No EPIC‐HIV	920/1792 (51.3)	*ref*		n/a	n/a	
Viral suppression (1 year after intervention)^b^						
Per arm						
CFI only	37/69 (53.6)	0.89 [0.74−1.07]	0.231	226/348 (64.9)	0.96 [0.77−1.21]	0.738
CFI + EPIC‐HIV	6/8 (75.0)	1.25 [0.85−1.85]	0.261	n/a	n/a	
EPIC‐HIV only	59/91 (64.8)	1.07 [0.82−1.38]	0.632	n/a	n/a	
Standard of care	145/239 (60.7)	*ref*		713/1050 (67.9)	*ref*	
Per CFI arms						
CFI	43/77 (55.8)	0.91 [0.76−1.09]	0.295	226/348 (64.9)	0.96 [0.77−1.21]	0.738
No CFI	204/330 (61.8)	*ref*		713/1050 (67.9)	*ref*	
Per EPIC‐HIV arms						
EPIC‐HIV	65/99 (65.7)	1.11 [0.88−1.40]	0.413	n/a	n/a	
No EPIC‐HIV	182/308 (59.1)	*ref*		n/a	n/a	

Note 1: *p*‐Values were calculated using Wald tests.

Note 2: Viral load results are corrected with a post‐stratification weight based on age and sex.

Abbreviations: ART, antiretroviral treatment; CFIs, conditional financial incentives; EPIC, Empowering People through Informed Choices for HIV; SoC, standard of care.

^a^ One hundred and sixty‐six individuals were excluded from the ART status because they were documented as dead or transferred out.

^b^ One year after the HITS intervention, viral load measurement was available for 407 men and 1398 women.

### 3.4 ART status 3 months after intervention

Among our initial study population of 2763 men and 7266 women living with HIV, 38 men and 128 women were excluded because they were documented as transferred out, died or migrated out of the surveillance survey area within the 3 months following HITS intervention. The Tier.Net data documented 63.4% (3632/5729) of individuals currently on ART (Appendix S5). Overall, 52.7% [50.1–55.2] (1435/2725) of men and 60.2% [58.2–62.1] (4294/7138) of women were documented on ART (Figure 2B). ART status before the intervention was similar between arms among men and women (global *p*‐value, *p* = 0.45 and *p* = 0.67, respectively).

Men and women in arms with CFI were not more likely to be on ART 3 months after the HITS intervention (Table 1). In the combined EPIC‐HIV arms analysis, men were not more likely to be on ART compared to the non‐EPIC‐HIV arms although the confidence interval was close to exclude the value one (RR 1.08 [0.99–1.17], *p* = 0.09).

### 3.5 VL suppression 1 year after the HITS visit

In 2018, 3668 HIV‐positive blood samples were collected for VL measurement. Of those, 518 samples were insufficient for the VL measurement test, leading to 3150 individuals (31.4% of the initial PLHIV included in our analysis) with a documented VL at trial completion. This sample was used to adjust the unbalanced distribution of sex and age for the 2019 sample (Appendix S1).

Among residents who consented to participate in the 2019 VL surveillance round, 2336 HIV‐positive blood samples were collected. Among them, 268 tests were excluded due to insufficient sample volume, 28 were duplicated and 235 did not belong to individuals from our pre‐trial baseline population leading to 1805 individuals (18.0% of the initial PLHIV included in our analysis) with a documented VL in 2019.

**Figure 3:**
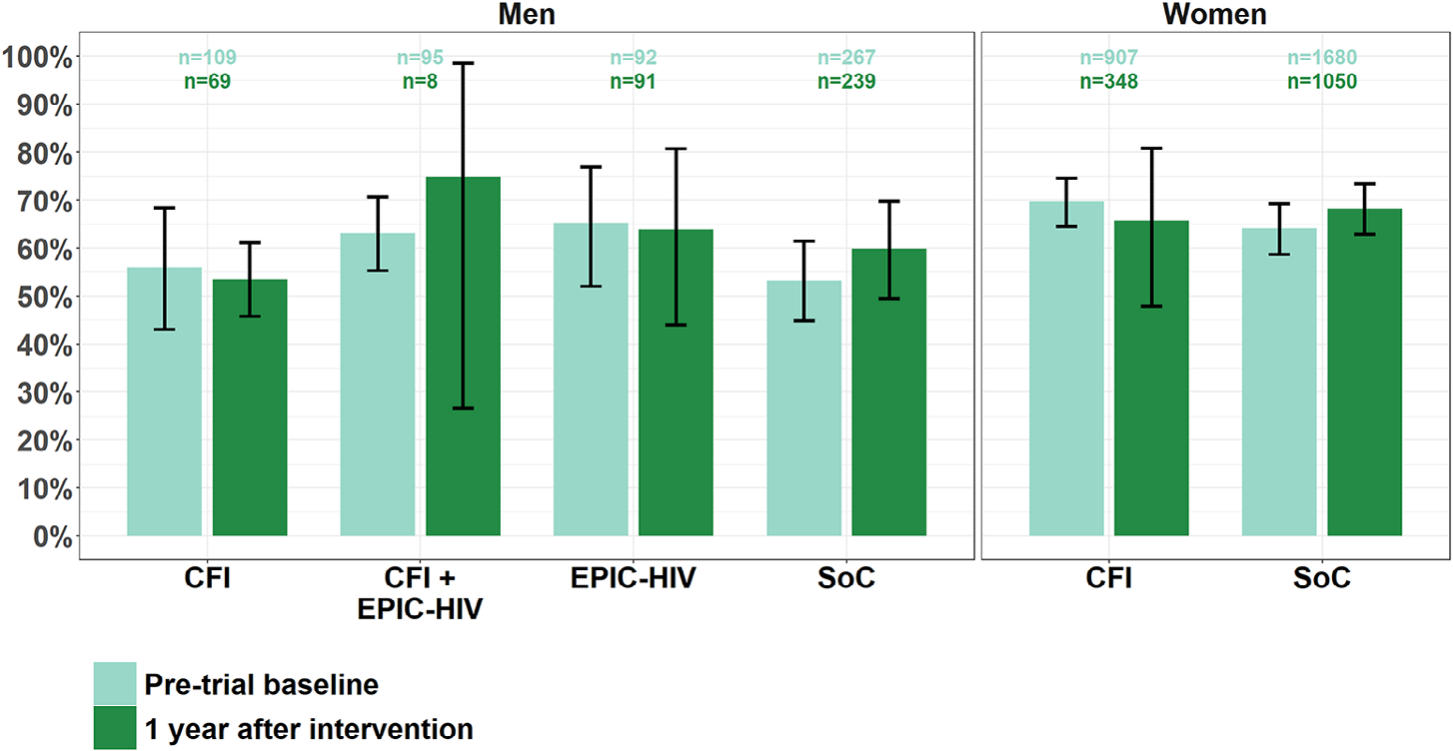
Percentage of men and women with viral load suppression per trial arm at pre‐trial baseline (*n* = 3150) and 1 year later (*n* = 1805).

Overall, 59.9% [53.2–66.0] (244/407) of men with valid samples and 67.6% [62.0–73.0] (945/1398) of women with valid samples were virally suppressed in 2019 (vs. 57.4% [52.2–62.0] (323/563) and 66.0% [62.2–70.0] (1708/2587) at pre‐trial baseline, Figure 3). No significant differences were observed for VL suppression between arms at pre‐trial baseline (global *p*‐value, *p* = 0.14 and *p* = 0.21 among men and women, respectively). The number of VL measurements collected in the combined intervention (i.e. CFI + EPIC‐HIV) arm in 2019 was particularly low compared to pre‐trial baseline (83 vs. 560 collected in 2018). Men in the EPIC‐HIV arms were associated but not significantly to viral suppression (RR 1.11 [0.88−1.40], *p* = 0.41) (Table 1). Neither men nor women in the CFI arms were significantly more likely to be virally suppressed 1 year after the intervention.

## 4 DISCUSSION

We have shown that a small CFI combined with home‐based HIV testing can contribute to increasing the number of men and women living with HIV aware of their HIV status. However, neither decision support application among men nor financial incentives seem to have influenced the number of people on ART or virally suppressed, respectively, 3 months and 1 year after the intervention.

Our findings demonstrate that CFIs not only lead to a higher number of individuals tested, but also result in the identification of more previously undiagnosed HIV cases [4, 5, 6]. Unlike previous studies on CFI, the strength of our methodology was to consider the overall impact of CFI on HIV status awareness at community level (i.e. considering both participants and non‐participants) and to consider previous HIV status awareness using data from a long‐established HIV surveillance cohort. Although the overall increase in the number of PLHIV who were aware of their HIV status and the associated effect size in the CFI arms may be seen as small, it has to be considered according to the high proportion of PLHIV already aware of their status before the trial; finding persons with undiagnosed HIV in this context is more difficult and is more likely to demand extensive effort and means [29]. In addition, increased HIV status awareness was observed despite the overall low participation in the annual HIV surveillance (i.e. less than half), which was a prerequisite for being offered to participate in the trial. Since its introduction in 2002, participation in the surveillance programme has steadily declined, likely due to study “fatigue” [30]. This suggests that introducing CFI for home‐based HIV testing uptake in a non‐surveillance area could lead to higher participation than our results indicate, and thus greater impact of the CFI on testing uptake and HIV status awareness. Therefore, CFI presents a promising approach for reaching the first 95%, even in settings with existing high HIV status awareness.

Neither the CFI nor the decision support application influenced the linkage to ART at 3 months. CFI focuses on the psychological leverage of an immediate reward when the future benefit of adopting a health behaviour is perceived as uncertain which can explain its impact on testing uptake and HIV awareness [31]. However, its non‐effectiveness on linkage to ART suggests that the perceived disadvantages of been linked to ART (e.g. fear of stigmatization if seen in an ART clinic) were not outweighed by the immediate reward; this result aligns with previous studies [7, 8, 9, 10]. On the other hand, the non‐effect of the decision support application was unexpected as many surveys documented counselling as an effective strategy to improve linkage to care [2, 12, 13]. In another paper, we report that linkage to care among men benefiting from the EPIC‐HIV was higher but only 12 months after the intervention, suggesting that EPIC‐HIV could reduce the time to be linked to care but not as fast as we anticipated in the study design [17]. Men newly diagnosed might need some time to “digest” the test result before taking the decision to link into care. In fact, during the co‐development of the EPIC‐HIV application with the local communities, the application was seen as encouraging men to link to HIV care. However, during the trial, interviews conducted as part of the process evaluation found that the app was perceived as insufficient as a standalone intervention without addressing other factors, such as the unwelcoming nature of health service clinics to men (e.g. men being treated poorly, waiting time, lack of privacy), individual experiences of HIV or emotional support [32].

Our study presents some limits. The HIV status was not documented for 24% of men and 12% of women residing in the study area which probably underestimates the total number of PLHIV. Some PLHIV may have been misclassified as not living with HIV because they acquired HIV between their last known HIV test the study start. These two previous considerations may have overestimated the number of PLHIV aware of their status or on ART. Our study used a higher VL suppression threshold (1550 copies/ml) than national guidelines (400 copies/ml) due to assay limitations, potentially overestimating true suppression in our population. ART coverage might have been underestimated due to residents seeking treatment outside the study area. However, because there were no differences in baseline characteristics after the randomization, it is unlikely that these selection bias would influence the effectiveness of the intervention—the selection bias being equivalent between arms.

A relatively low number of men living with HIV used the EPIC‐HIV application (only 122 and 14 men completing the EPIC‐HIV‐1 and EPIC‐HIV‐2 application, respectively) which might explain the lack of impact of the EPIC‐HIV application on ART linkage and viral suppression. In addition, the collection of blood samples for VL measurement 1 year after the trial was particularly low in EPIC‐HIV arms, especially the combined one. The data collection in the combined arm, unfortunately, coincided with the celebration of an important religious event involving the travel of numerous residents. Thus, our results relative to the impact of EPIC‐HIV on ART linkage and VL remain inconclusive and call for further evidence.

CFI‐only increased HIV status awareness in men without significantly impacting awareness in the combined intervention arm or in the factorial analysis. Notably, despite similar testing rates, the CFI‐only arm yielded more positive rapid tests (41 vs. 22). Potential explanations include residual cluster heterogeneity in HIV incidence or awareness among men at baseline.

Our results contribute to filling the research gap on the potential impact of CFI and digital application on the HIV care cascade. Scaling up a combined CFI with home‐based HIV testing could be considered in other high‐prevalence settings with low HIV awareness. However, in such situations, additional interventions that facilitate linkage to care, such as peer navigation of PLHIV to clinic, would be necessary due to the ineffectiveness of CFI on linkage to care [33]. While our results might be applicable to other similar settings in Africa, combining CFI with another delivery model of HIV testing might not yield the same outcomes on HIV awareness. For example, combining CFI with person‐initiated testing might encourage more frequent testing than necessary among individuals with low HIV exposure. Questions also remain regarding testing uptake changes after CFI is no longer available. Some individuals might be less likely to undergo testing “for free” if it was previously paid for. The cost‐effectiveness of such a strategy and the optimal implementation method (e.g. frequency, targeting) also require further assessment.

While we found no effect of the application on the HIV care cascade, our results do not definitively disprove the potential of digital strategies to improve the cascade. mHealth applications remain particularly complex due to the numerous design decisions involved, each of which can affect user engagement and the intervention's influence. Our study only explored a single, specific application, which cannot represent the full potential of digital applications.

Although CFIs and digital applications have the potential to improve access to care, their effectiveness is limited as standalone interventions. In contexts of income insecurity and low access to basic infrastructure, a one‐off CFI or digital application intervention will not address the wider determinants of health and health‐seeking behaviour, which are likely stronger influences on decisions to link to care [34, 35]. Thus, CFIs and digital applications should be implemented alongside other interventions that address those wider determinants.

## 5 CONCLUSION

Small CFIs combined to home‐based HIV testing increase the number of PLHIV aware of their status at a community level. However, neither CFI nor EPIC‐HIV was sufficient to increase the number of people on ART at 3 months. Due to the low sample size in the EPIC‐HIV arms, additional evidence is needed to confirm the impact of EPIC‐HIV alone and the combination of both CFI and EPIC‐HIV on viral suppression.

## COMPETING INTERESTS

All authors declare: no support from any organization for the submitted work; no financial relationships with any organization that might have an interest in the submitted work in the previous 3 years, no other relationships or activities that could appear to have influenced the submitted work. In addition, none of the authors have current or previous professional affiliation with the funder.

## AUTHORS’ CONTRIBUTIONS

FT and TB are the principal investigators and developed the study design and protocol in collaboration with MS, SW, NM, JS, HMY and AD. TM and DG contributed to the implementation of the study. SW, PM, OA, TM, TZ and JS designed the content of EPIC‐HIV and AB oversaw its development into app‐based delivery. OA, JS and MS led the evaluation of the trial particularly the application and the financial incentives. H‐YK and DG cleaned and prepared the data. MI analysed the data and wrote the first draft of the manuscript. MI and H‐YK had full access to all the data and take responsibility for the integrity of the data as well as the accuracy of the data analysis. All authors provided substantive feedback on the manuscript and have read and approved the final version. MI is the guarantor. The corresponding author attests that all listed authors meet authorship criteria and that no others meeting the criteria have been omitted.

## FUNDING

The research is funded by the National Institute of Allergy and Infectious Diseases (NIAID) of the National Institutes of Health (NIH) under Award Number R01AI124389 (PIs: Frank Tanser and Till Bärnighausen). EPIC‐HIV development was supported by the Engineering and Physical Sciences Research Council (EPSRC) Interdisciplinary Research Collaboration (IRC) Early‐warning Sensing Systems for Infectious Diseases (i‐sense) EP/K031953/1 and MRC MR/P024378/1. Frank Tanser and Till Bärnighausen are also supported by the NIH grant (R01‐HD084233). The Africa Health Research Institute's Health and Demographic Surveillance Information System is funded by the Wellcome Trust (201433/A/16/A), and the South Africa Population Research Infrastructure Network (SAPRIN: funded by the South African Department of Science and Technology and hosted by the South African Medical Research Council).

## DISCLAIMER

The content is solely the responsibility of the authors and does not necessarily represent the official views of the funding bodies. Frank Tanser and Till Bärnighausen are also supported by the NIH grant (R01‐HD084233). Nuala McGrath is a recipient of an NIHR Research Professorship award (RP‐2017‐08‐ST2‐008). Maryam Shahmanesh is a recipient of an NIHR Research Professorship award (NIHR 301634). The corresponding author confirms having access to all the data in the study and had final responsibility for the decision to submit for publication. The corresponding author affirms that the manuscript is an honest, accurate and transparent account of the study being reported; that no important aspects of the study have been omitted; and that any discrepancies from the study as planned (and, if relevant, registered) have been explained.

## TRIAL REGISTRATION

ClinicalTrials.gov NCT03757104.

## Supporting information


**Appendix S1**. Characteristics of individuals tested for HIV viral load in 2018 and 2019, Unweighted data.
**Appendix S2**. Flow diagram for the HITS trial for the rapid testing uptake and linkage to care within 6 weeks.
**Appendix S3**. HIV status awareness among HIV‐positive men and women, 2018, with mid‐point method for estimating the date of last HIV test (*n* = 10,029).
**Appendix S4**. Risk factors for HIV status awareness and linkage to ART following HITS visit among HIV‐positive men and women, 2018−2019, with mid‐point method for estimating the date of last HIV test (*n* = 10,029).
**Appendix S5**. Frequency of the source of the ART status documentation per arm among those documented as on ART, before the HITS visit date (A) and at the end of the HITS visit date (B).

## Data Availability

The de‐identified datasets are available upon reasonable request through the Africa Health Research Institute (AHRI) data repository at https://data.ahri.org/index.php/home
