# Supplementary material for: Population impacts of conditional financial incentives and a male‐targeted digital decision support application on the HIV treatment cascade in rural KwaZulu Natal: findings from the HITS cluster randomized clinical trial
Source: J Int AIDS Soc. 2024 May 2;27(5):e26248. doi: 10.1002/jia2.26248 (PMC11063775; doi:10.1002/jia2.26248)
Supplement: Supplementary file 1 — Appendix S1. Characteristics of individuals tested for HIV viral load in 2018 and 2019, Unweighted data. Appendix S2. Flow diagram for the HITS trial for the rapid testing uptake and linkage to care within 6 weeks. Appendix S3. HIV status awareness among HIV‐positive men and women, 2018, with mid‐point method for estimating the date of last HIV test (n = 10,029). Appendix S4. Risk factors for HIV status awareness and linkage to ART following HITS visit among HIV‐positive men and women, 2018−2019, with mid‐point method for estimating the date of last HIV test (n = 10,029). Appendix S5. Frequency of the source of the ART status documentation per arm among those documented as on ART, before the HITS visit date (A) and at the end of the HITS visit date (B). [file JIA2-27-e26248-s001.docx]

Extended Data

**Population impacts of conditional financial incentives and a male-targeted digital decision support application on the HIV treatment cascade in rural KwaZulu Natal: Findings from the HITS cluster randomized clinical trial.**

[Appendix 1. characteristics of individuals tested for HIV viral load in 2018 and 2019, Unweighted data. 2](#_Toc159254690)

[Appendix 2. Flow diagram for the HITS trial for the rapid testing uptake and linkage to care within 6 weeks. 3](#_Toc159254691)

[Appendix 3. HIV status awareness among HIV positive men and women, 2018, with mid-point method for estimating the date of last HIV test (n=10,029). 4](#_Toc159254692)

[Appendix 4. Risk factors for HIV status awareness and linkage to ART following HITS visit among HIV positive men and women, 2018-2019, with mid-point method for estimating the date of last HIV test (n=10,029). 5](#_Toc159254693)

[Appendix 5. Frequency of the source of the ART status documentation per arm among those documented as on ART, before the HITS visit date (A) and at the end of the HITS visit date (B). 6](#_Toc159254694)

Appendix 1. characteristics of individuals tested for HIV viral load in 2018 and 2019, Unweighted data.

|  | | **2018**  (n=3,150) | | **2019**  (n=1,805) | **p-value** |
| --- | --- | --- | --- | --- | --- |
| **Sex** | |  |  | | <0.001 |
|  | Male | 563 (18%) | 407 (22%) | |  |
|  | Female | 2,587 (82%) | 1,398 (78%) | |  |
| **Age** | |  |  | | <0.001 |
|  | 15-24 | 375 (12%) | 150 (8%) | |  |
|  | 25-34 | 898 (29%) | 425 (23%) | |  |
|  | 35-44 | 811 (26%) | 528 (29%) | |  |
|  | 45-54 | 570 (18%) | 396 (22%) | |  |
|  | 55 and more | 496 (16%) | 306 (17%) | |  |
| **Arm** | |  |  | | 0.069 |
|  | CFI only | 551 (17%) | 342 (19%) | |  |
|  | CFI + EPIC-HIV | 560 (18%) | 83 (5%) | |  |
|  | EPIC-HIV only | 548 (17%) | 404 (22%) | |  |
|  | Standard of care | 1,491 (47%) | 976 (54%) | |  |

ART: antiretroviral treatment, CFI: Conditional financial incentives, EPIC: Empowering People through Informed Choices for HIV

Note: p-values were calculated using Wald tests.


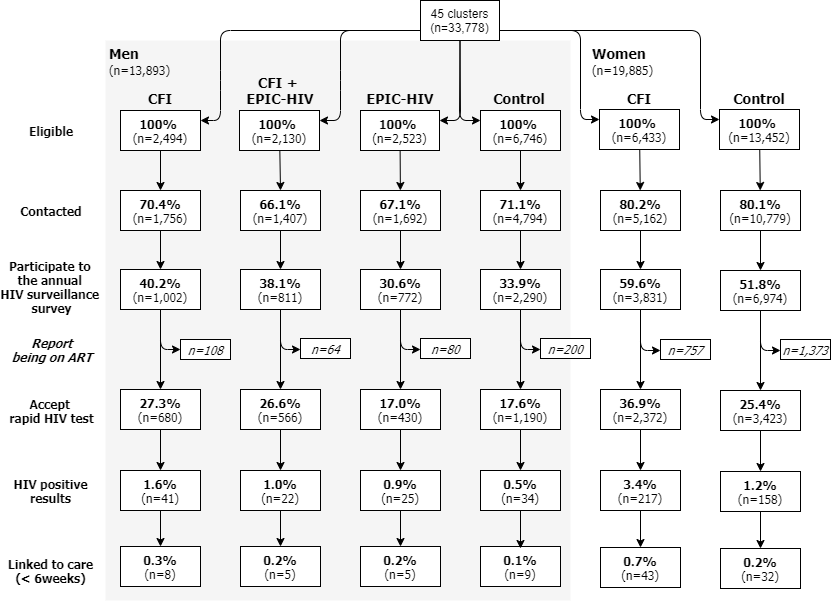


Appendix 2. Flow diagram for the HITS trial for the rapid testing uptake and linkage to care within 6 weeks.

CFI: conditional financial incentive, EPIC-HIV: empowering people through informed choices for HIV


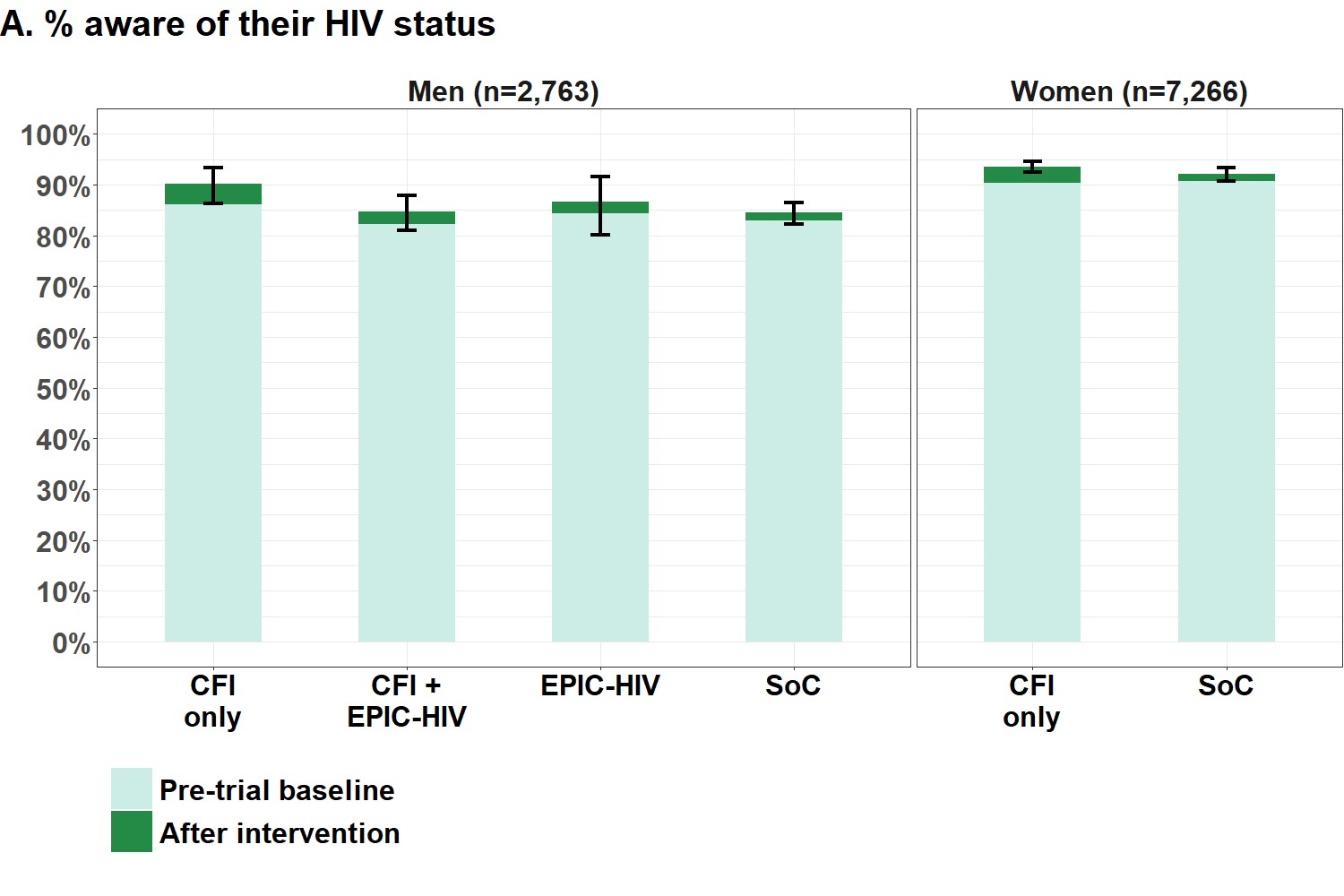


Appendix 3. HIV status awareness among men and women living with HIV, 2018, with mid-point method for estimating the date of last HIV test (n=10,029).

CFI: conditional financial incentive, EPIC-HIV: empowering people through informed choices for HIV, SoC: standard of care.

Note 1: 95% confidence interval are computed for the HIV status awareness at the end of the HITS visit date

Appendix 4. Risk factors for HIV status awareness and linkage to ART following HITS visit among men and women living with HIV, 2018-2019, with mid-point method for estimating the date of last HIV test (n=10,029).

|  |  | **Men (n=2,763)** | | | **Women (n=7,266)** | | |
| --- | --- | --- | --- | --- | --- | --- | --- |
|  | | n/N (%) | Relative Risk (95% CI) | p-value | n/N (%) | Relative Risk (95% CI) | p-value |
| **HIV status awareness** | | |  |  |  |  |  |
|  | **Intervention group analysis** | |  |  |  |  |  |
|  | CFI only | 444/492 (90.2) | 1.07 [1.03-1.11] | 0.001 | 2266/2421 (93.6) | 1.02 [1.00-1.04] | 0.059 |
|  | CFI + EPIC-HIV | 352/416 (84.6) | 1.00 [0.96-1.04] | 0.925 | n/a | n/a |  |
|  | EPIC-HIV only | 459/530 (86.6) | 1.03 [0.97-1.09] | 0.383 | n/a | n/a |  |
|  | Standard of care | 1119/1325 (84.5) | *ref* |  | 4460/4845 (92.1) | *ref* |  |
|  | **Factorial analysis** | |  |  |  |  |  |
|  | CFI | 796/908 (87.7) | 1.03 [0.99-1.07] | 0.116 | 2266/2421 (93.6) | 1.02 [1.00-1.04] | 0.059 |
|  | No CFI | 1578/1855 (85.1) | *ref* |  | 4460/4845 (92.1) | *ref* |  |
|  | **Factorial analysis** |  |  |  |  |  |  |
|  | EPIC-HIV | 811/946 (85.7) | 1.00 [0.96-1.04] | 0.868 | n/a | n/a |  |
|  | No EPIC-HIV | 1563/1817 (86.0) | *ref* |  | n/a | n/a |  |

ART: antiretroviral treatment, CFI: Conditional financial incentives, EPIC: Empowering People through Informed Choices for HIV

Note: p-values were calculated using Wald tests.


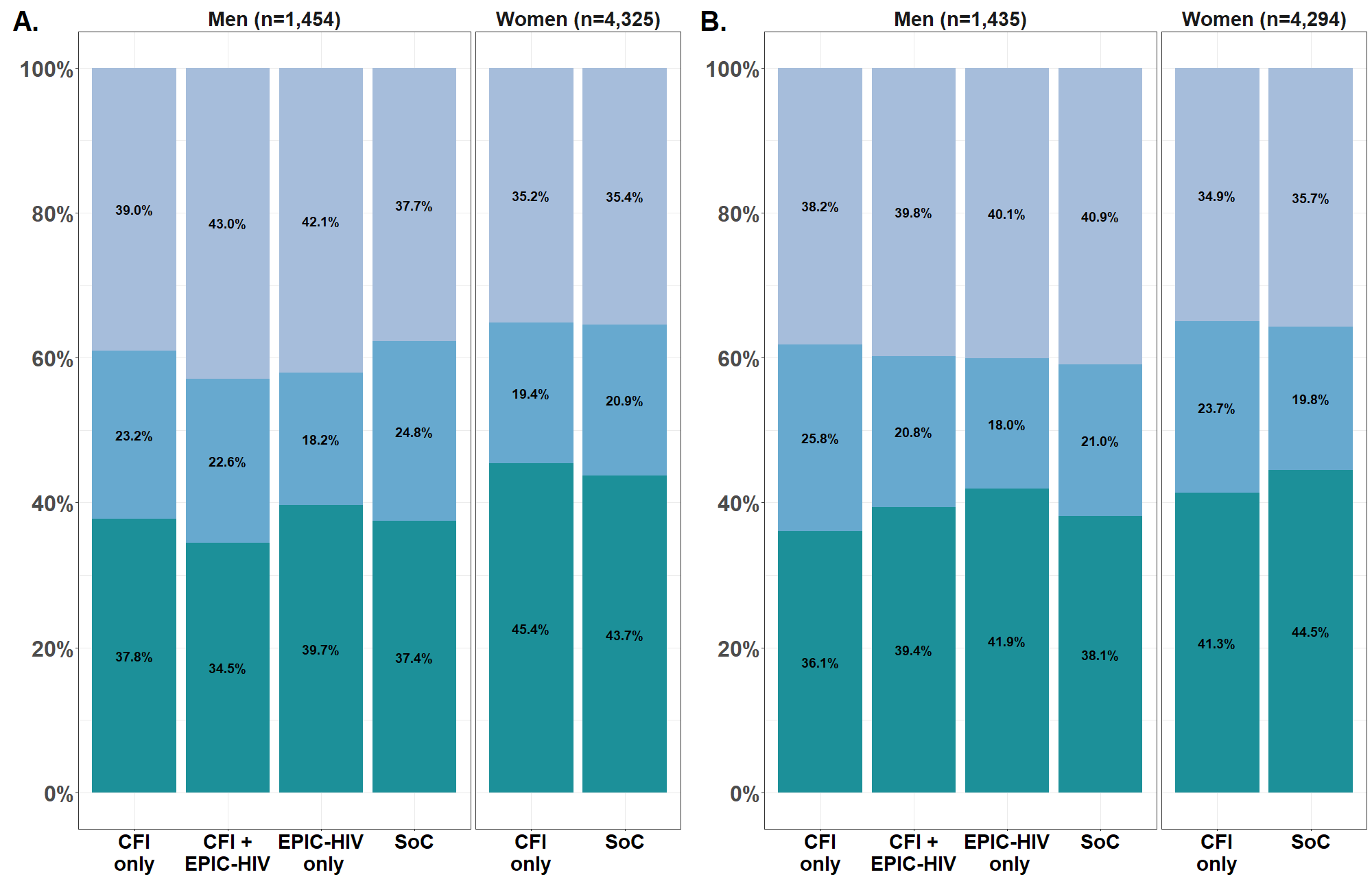


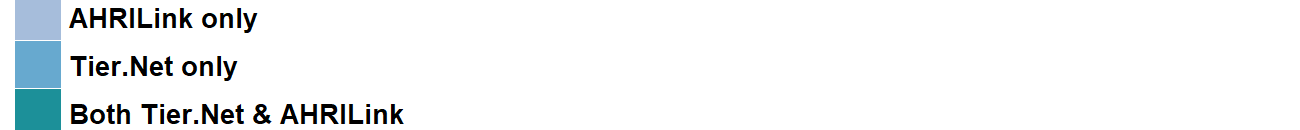


Appendix 5. Frequency of the source of the ART status documentation per arm among those documented as on ART, at pre-trial baseline (A) and three months after the intervention (B).

CFI: conditional financial incentive, Clinic Link: patient electronic record system to the 11 clinics located in the study area, EPIC: empowering people through informed choices for HIV, HITS: home-based intervention to test and start, SoC: standard of care, Tier.Net: electronic record system of patients enrolled in the local public HIV Treatment and Care Programme
